# PbI_2_ Passivation of Three Dimensional PbS Quantum Dot Superlattices Toward Optoelectronic Metamaterials

**DOI:** 10.1021/acsnano.4c04076

**Published:** 2024-07-02

**Authors:** Jacopo Pinna, Elisa Pili, Razieh Mehrabi Koushki, Dnyaneshwar S. Gavhane, Francesco Carlà, Bart J. Kooi, Giuseppe Portale, Maria Antonietta Loi

**Affiliations:** † Zernike Institute for Advanced Materials, University of Groningen, Nijenborgh 4, Groningen 9747 AG, The Netherlands; ‡ Diamond House, Harwell Science and Innovation Campus, Diamond Light Source Ltd, Didcot, Oxfordshire OX11 0DE, United Kingdom

**Keywords:** colloidal quantum dots, superlattice, self-assembly, mobility, passivation

## Abstract

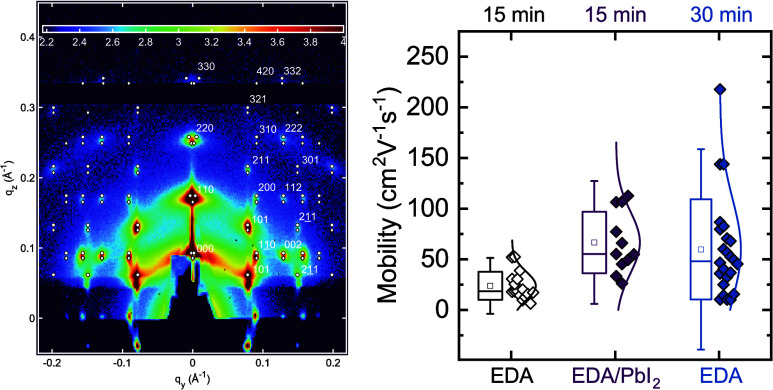

Lead chalcogenide colloidal quantum dots are one of the most promising materials to revolutionize the field of short-wavelength infrared optoelectronics due to their bandgap tunability and strong absorption. By self-assembling these quantum dots into ordered superlattices, mobilities approaching those of the bulk counterparts can be achieved while still retaining their original optical properties. The recent literature focused mostly on PbSe-based superlattices, but PbS quantum dots have several advantages, including higher stability. In this work, we demonstrate highly ordered 3D superlattices of PbS quantum dots with tunable thickness up to 200 nm and high coherent ordering, both in-plane and along the thickness. We show that we can successfully exchange the ligands throughout the film without compromising the ordering. The superlattices as the active material of an ion gel-gated field-effect transistor achieve electron mobilities up to 220 cm^2^ V^–1^ s^–1^. To further improve the device performance, we performed a postdeposition passivation with PbI_2_, which noticeably reduced the subthreshold swing making it reach the Boltzmann limit. We believe this is an important proof of concept showing that it is possible to overcome the problem of high trap densities in quantum dot superlattices enabling their application in optoelectronic devices.

Since their first discovery, semiconducting colloidal quantum dots (CQDs) have demonstrated tremendous potential in all subfields of optoelectronics owing to their unique optical properties stemming from the quantum confinement effect. Photodetection in the short-wavelength infrared (SWIR) range could particularly benefit from the implementation of CQDs, as the industrial standards in this sector are dominated by traditional bulk semiconductors like InGaAs, which contain rare elements (Ga) and are grown in extreme thermodynamical conditions. As a result, the technology is poorly scalable and very costly. The consumer market applications that are seeing an increasing demand for cheaper SWIR detectors are night vision applications and light detection and ranging (LiDAR) systems in the automotive sector.^1^

Among available CQDs active in the SWIR, the lead chalcogenide family represents an ideal candidate for these applications. First, the bandgap tunability in a wide wavelength range (800–3000 nm)^2−4^ enables targeting specific applications on demand. Second, the synthesis, processing, and deposition from solution have the potential to make it cheap and scalable. Lastly, the quantum-confined nature of the CQDs ensures minimal leakage current even at room temperature. However, poor electrical conductivity is generally reported due to the disordered nature of CQDs’ thin films and their high density of trap states.^5^ In the best examples, charge mobilities below 1 cm^2^ V^–1^ s^–1^are obtained, which are several orders of magnitude less than traditional bulk semiconductors.^6−10^ A way to overcome the problem of moderate mobilities is to assemble the CQDs in ordered arrays to form the so-called superlattices (SLs).^11,12^ Several theoretical works have predicted that such CQD solids should display high mobilities comparable to the bulk counterparts and more exotic electronic properties like Dirac cones for the honeycomb lattice.^13−19^

Superlattices with various geometries, structural features, and degree of ordering have been fabricated both with PbSe and PbS CQDs.^20−29^ While PbSe has been historically preferred because of showcasing higher mobilities in the bulk and easiness of synthesis with higher monodispersity, in the CQDs counterpart, its chemistry makes it particularly susceptible to oxidation. Lead sulfide instead shows better stability and is ubiquitously employed for most SWIR optoelectronic applications nowadays. However, research has always focused on disordered films and their chemical functionalization leaving unexplored the ordered arrays. Earlier reports on PbS superlattices have focused on structural characterization in systems with the native oleic acid ligands, often studying the crystallization upon controlled drying.^30−35^ While these samples displayed excellent ordering, efficient tuning of their thickness (usually around 120 nm) has never been demonstrated. However, the presence in these superlattices of the insulating oleic acid ligands constitutes a fundamental limitation for their device applications. Attempts of solid-state ligand exchange on SLs proved to be particularly detrimental, giving rise to highly defective materials and limiting their use in devices.^31^ As a consequence, little to no attention has been dedicated to measuring the transport properties of these PbS CQD superlattices.^36,37^

Highly ordered superlattices of the CQDs can be obtained by assembling them on a liquid–liquid or liquid–vapor interface and subsequently removing the ligands to induce epitaxial necking of specific facets.^38−41^ Most studies have focused on 2D SLs that usually have a square geometry. With the usage of an ionic liquid gating in a field-effect transistor (FET), electron mobilities up to 15 cm^2^ V^–1^ s^–1^ have been recently reported for PbS CQDs, values which are on par with analogous 2D PbSe SLs (13–24 cm^2^ V^–1^ s^–1^).^22,29,42,43^ We recently demonstrated electron mobilities up to 270 cm^2^ V^–1^ s^–1^ in 3D PbSe CQDs SLs with analogous ionic gel-gated FETs, owing to a significantly improved ordering with respect to the 2D systems. This fact motivated us to study 3D superlattices composed of PbS CQDs, given the higher interest and versatility for technological applications. In fact, the possibility of using 3D SLs for SWIR photodetection has been barely investigated,^44^ and tuning the thickness is an important necessary step to build this future technology.

In this work, we demonstrate the self-assembly of commercially available PbS quantum dots, with state-of-the-art quality and monodisperity, into 3D SLs with tunable thicknesses up to 220 nm. We show that it is possible to achieve an efficient and complete ligand exchange even with such thick structures, while still retaining the optical properties of the building blocks. These samples have excellent ordering in all three spatial dimensions as demonstrated by the high in-plane and out-of-plane coherence lengths. Finally, we implement these thick 3D SLs in ion gel-gated FETs, which allow us to study the intrinsic properties of the CQD solid upon filling of trap states up to a surface density of 10^13^ cm^–2^. These transistors show n-type behavior with excellent on/off ratio, high conductance, and reproducibility over 50 devices. We find that long (30 min) ligand exchange treatments lead to electron mobilities up to 220 cm^2^ V^–1^ s^–1^, but large variations within samples. Reducing the ligand exchange time (15 min) significantly narrows the distribution, but mobilities are dramatically decreased while the surface trap density further increases. Here, we demonstrate that by treating the superlattices with PbI_2_, we recover the average mobility reaching up to 67 cm^2^ V^–1^ s^–1^. Most importantly, this treatment significantly reduces the surface trap density as proven by average subthreshold swings decreasing from 80 ± 8 to 68 ± 6 mV/dec with several devices at the Boltzmann limit level.

## Results and Discussion

**Figure 1:**
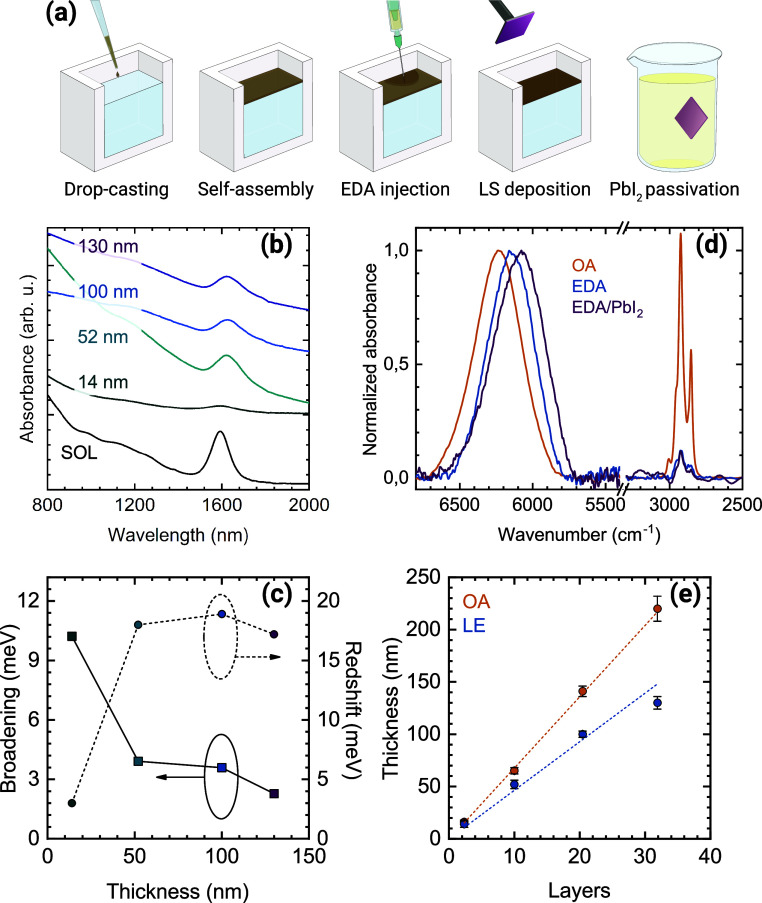
(a) Schematics of the 3D PbS superlattice’s fabrication procedure. (b) Comparison between the absorbance spectrum of the PbS colloidal solution (SOL) and EDA-treated SLs of increasing thickness. Curves are offset for clarity; (c) broadening and redshift of the first excitonic peaks for the same samples as in (b). The values are extracted from a Gaussian fitting of the absorption spectra, and they are indicated with respect to the solution values; (d) normalized transmission FTIR spectra of 20-layers SLs with native ligands (OA), EDA-treated (EDA), and after postdeposition passivation with PbI_2_ in DMSO (EDA/PbI_2_); (e) thickness of the SLs films with native ligands and after EDA treatment (LE) as measured by AFM. The dashed lines are linear fits meant to be used as a guide for the eye.

For our experiments, we chose commercially available PbS QDs with a diameter of 6.2 ± 0.2 nm. The first excitonic peak, as measured in the colloidal solution in octane, is at 1590 nm (Figure 1a), thus ideal for telecommunication applications and photodetection in the SWIR. The size dispersity of only 4% makes these materials especially suitable for self-assembly and it also translates to a very limited energetic disorder (σ/*E*_0_ = 2.5%), which is known to be fundamental for charge transport efficiency.^12,45^

We fabricated three-dimensional superlattices of different thicknesses following the method reported in our recent work (Figure 1a).^46^ Briefly, a concentrated colloidal solution dispersed in octane is drop-casted at the center of a PTFE bath filled with ethylene glycol (EG), which serves as a spreading subphase. Due to the difference in surface energy between octane and EG, the CQD solution quickly spreads through the whole surface while the solvent slowly evaporates due to its high boiling point (125.6 °C). The thickness of the films can be tuned based on the volume of solution from mono/bilayer up to around 220 nm.

Once the solvent is fully evaporated, the CQDs have self-assembled in an SL but are still covered with the native oleic acid (OA) ligands (*vide infra*). To obtain a film that is suitable for optoelectronic applications, we remove these ligands by injecting an ethylenediamine (EDA) solution directly into the EG subphase. We call this process ligand exchange (LE) for coherency with the literature. The exchange reaction is different depending on the involved facets of the CQDs: the {100} ones undergo ligand stripping while for the {111} ones, a mechanism has been proposed recently.^47^

Compared to the case of the more commonly used PbSe, the LE in PbS is less tumultuous and a minimum of 15 min is required for the reaction to occur in thicker films. The ligand stripping causes a visible darkening in the floating film, which also becomes more reflective. Usually, this is associated with the reduced interdot distance and consequent increased density and volume shrinking. Several works have reported that lead chalcogenide CQDs form epitaxial necks through their {100} facets because of this ligand stripping.^22,41,48−50^ At the end of the LE, the obtained epitaxially connected superlattices are transferred to the substrate of choice via Langmuir–Schaefer (LS) deposition. To passivate traps known to occur after this type of ligand exchange or removal, we further immerse the deposited samples in a PbI_2_ solution in DMSO. In the case of charge transport measurements, the substrates also undergo annealing for 20 min at 120 °C to remove residual solvents.

To assess the energetic landscape of the LE SLs, we performed absorption measurements on the EDA-treated samples with thicknesses ranging from 14 to 130 nm (Figure 1b) from which we determined both the broadening and the redshift of the first excitonic peak. The spectra clearly show that quantum confinement is retained in all cases, demonstrating that the EDA treatment does not cause excessive neck growth and sintering. The most significant broadening of the excitonic peak, measured as the σ of the best-fitting Gaussian, is found in the thinnest sample. The broadening value decreases with increasing film thickness down to merely 2 meV (with respect to the pristine CQDs solution) in the thickest sample. This observation is in line with our previous experiment with PbSe CQDs, where the increased energetic disorder of the quasi-2D samples is due to the higher structural disorder. On the other hand, the redshift grows from 3 meV to about 18 meV with increasing thickness (Figure 1c). A change in the position of the excitonic peak is associated with the CQDs’ size and dielectric environment. In this case, the higher redshift is present in all the 3D SLs, and it might be due to the formation of epitaxial necks in all six {100} facets, leading to a higher delocalization of the electronic wave function.

To check the influence of the thermal treatment on the confinement, we measured the absorption spectrum of a 100 nm-thick sample after annealing. We found that the excitonic peak is preserved, but as expected the absolute broadening has now increased to 32 meV and the redshift is 39 meV (Figure S1). Therefore, the increase in broadening and redshift is a clear indication of enhanced connectivity and epitaxial growth of the necks resulting in wave function delocalization. Calculations have shown that in 2D square PbSe SLs, depending on the neck width to quantum dot diameter ratio, the fwhm of the first excitonic transition can increase up to 97 meV owing to the bandwidth of the formed minibands.^19^

To gain insights into the LE efficiency, we performed FTIR absorption measurements on SL fabricated in three different conditions: a pristine film with the native OA; an EDA-treated film; a third film like the second one but further passivated with PbI_2_ and annealed at 120 °C (Figure 1d). We use the excitonic peak (broad feature at high wavenumbers), which is preserved in all curves, to normalize the IR spectra after background correction. The redshift obtained with these measurements is in agreement with the NIR-Vis ones while no significant broadening was observed. The C–H vibrations are visible at about 2900 cm^–1^, and the OA sample shows intense and narrow peaks that match the vibrational signatures of oleic acid (Figure S2). Additionally, absorptions at 1400 and 1540 cm^–1^ that correspond to the carbon-oxygen double bond are also evident.

The EDA treatment is proven by the strong suppression of all the peaks in the −CH region. At such low intensity, the major C–H bands are still distinguishable except for the 3006 cm^–1^ one (alkene C–H stretching on the OA), which might be absent or not detectable due to the poor signal-to-noise ratio (SNR) of this spectrum. The fraction of removed oleate ligands is determined to be about 83% by considering the ratio of the total C–H peak area in the two spectra. However, we noted that the peaks’ relative heights to each other are significantly different before and after ligand exchange. This could be ascribed to the residual presence of EG or EDA in the LE film despite acetonitrile washing and overnight vacuum drying. As EG and EDA share the C–H signals with native OA, it becomes rather challenging to determine the OA removal fractions at the current SNR. It is clear though that a residual organic fraction could not be removed from the samples. The C=O peaks would be discriminant in this case, but we could not observe them in the EDA sample. Since peaks originating from this bond are usually very strong and considering the absence of the alkene C–H peak, it is possible that oleic acid is mostly removed, and the organic fraction has a different origin. If a significant part of the C–H signal is due to residual EG trapped in the SL structure (bound or not), its removal solely with temperature or vacuum would be rather challenging due to its high boiling point. We also could not observe any features deriving from the coverage of EDA or ethylene glycoxide on the CQDs facets that were recently observed in 3D PbSe SLs, as neither N–H nor O–H signals are detectable.^47,50^ The passivation treatment with PbI_2_ and subsequent annealing does not seem to have a strong influence on the FTIR spectra of the sample, except for a further reduction of the peaks’ intensity suggesting further removal of organic residuals from the CQDs surface.

Given the relevance for technological applications, we formed SLs with thicknesses above 200 nm. This is particularly important for photodetection since the absorption increases exponentially with the film thickness. Up until now, CQD SLs in the literature are always 2D owing to their expected exotic electronic properties,^13,14^ and such PbS 3D SLs have never been fabricated. To accurately measure the thickness of the deposited film, we performed AFM on samples from 3 to 32 layers both with native OA ligands and after LE (Figure 1e). The SLs with the native ligands show that the thickness linearly increases with the volume of the colloidal solution used. The set of samples spans from 14 to 220 nm in thickness. The LE SLs instead show a systematic decrease in thickness which seems to deviate from linearity, particularly for the thickest sample. Considering that the LE samples are directly transformed from the OA ones, the reduction in thickness is to be expected considering the removal of the ligands which are approximately 2 nm in length.

**Figure 2:**
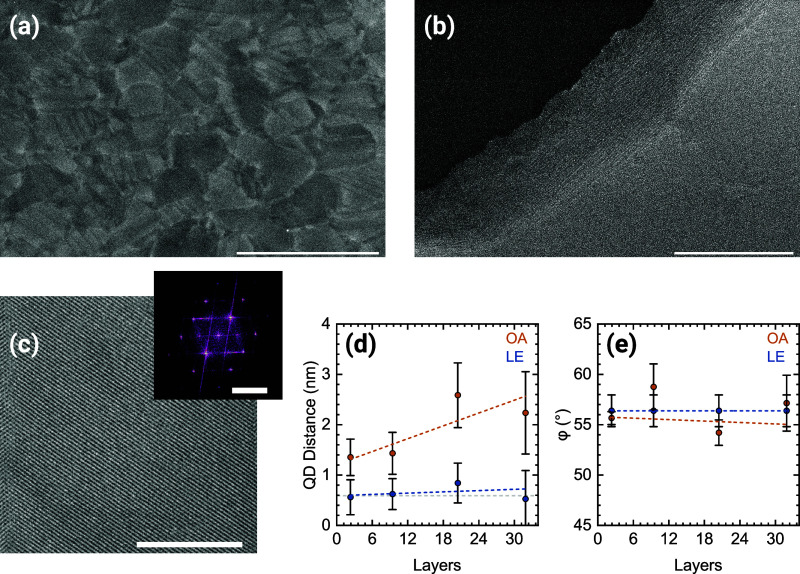
Electron microscopy structural investigation. (a) Low-magnification STEM-HAADF micrograph of the thickest OA sample (scale bar: 10 μm); (b) SE micrograph of the same sample at the edge of a macroscopic crack. The sharp edge of the SL domain enables us to see the ordered stacking of the CQD layers (scale bar: 500 nm); (c) high-magnification STEM-HAADF micrograph of the sample in panels (a) and (b) showing highly ordered single-crystalline SL domain. The inset FFT shows sharp reflections that point to a {110}_SL_ BCC unit cell (scale bars: 200 nm, 0.2 nm^–1^); (d) distance between neighboring CQD and (e) angle between the main crystallographic axes of the SL as a function of the number of layers for both oleic acid and ligand exchanged samples. The colored dashed lines are a guide to the eye while the gray dashed line indicates the PbS lattice parameter and is used as reference.

These 3D superlattices present several structural features over different length scales that are well evidenced by electron microscopy characterization (Figure 2). Since the noticeable thickness is an issue for electron transmission, we used a combination of scanning-TEM high-angle annular dark-field detection (STEM-HAADF) and secondary electron (SE) imaging to characterize the superficial parts of the thickest samples.

In Figure 2a, we present a STEM-HAADF micrograph of a 220 nm-thick OA-SL with a large field of view (30 × 20 μm^2^). We noticed that even at these large scales, the samples displayed excellent coverage with almost no pinholes nor nanoscaled cracks. The SL domains are clearly visible and span from several hundreds of nanometers to a few micrometers. The homogeneous contrast indicates a uniform thickness through the field of view, which is also confirmed by AFM measurements (Figure S3). The measured roughness over a 10 × 10 μm^2^ area is 3.7 ± 0.7 nm confirming that adjacent grains in a mesoscopic region all have the same thickness with a deviation of approximately one QD layer. Another interesting feature of these samples is the frequent occurrence of twinned grains with a specific and reproducible angle of around 108° (Figure S4). This might be an indication that during solvent evaporation and consequent SL grains nucleation and crystallization, adjacent domains tend to follow preferential in-plane crystallographic orientations contributing to the overall ordering of the superstructure.

Since there is no previous example in the literature of lead chalcogenide CQD SLs grown to such thicknesses, it is of great interest to understand if the structure remains coherent along the out-of-plane (OP) direction. By performing SE imaging along macroscopic cracks, we could clearly distinguish the several layers of the SLs and the single CQDs (Figure 2b). As revealed by the fast Fourier transform (FFT) on the edge of the domains, the coherent ordering appears as preserved also in the vertical direction (Figure S5). While this observation is very promising, a more accurate experimental technique needs to be used to confirm this observation (*vide infra*).

The in-plane ordering, geometry, and CQD nearest-neighbor distance can be determined from STEM-HAADF micrographs of single-crystalline domains (Figure 2c). The OA samples appear to all have a similar structure with the CQDs disposed on parallel chains. Along these chains, the nanocrystals occupy alternating positions over two partially overlapping planes in the vertical direction. The FFT of such regions shows that the samples are highly ordered as is evident from the sharp reflections in the pattern. The geometry of the FFT is recurrent in these types of samples and suggests a body-centered cubic (BCC) type of unit cell with the {110}_SL_ planes parallel to the substrates.^33,34,46,50,51^

For clarity purposes, all the structural analysis reported from this point will focus on samples where the LE duration was 30 min, and no postpassivation was performed. From the FFTs of single-crystalline domains, we determined the nearest-neighbor (NN) distance between CQD on the same plane and the angle φ between the two main SL crystallographic directions for the systematic series of samples from 3 to 32 layers before (OA) and after LE (Figure 2d,e). To obtain the distance between the CQDs, we subtracted their size from the NN distance. For the OA samples, we observed distances in the range of 1.4 to 2.6 nm, compatible with the presence of two ligand shells partially interpenetrated. As expected, when LE is performed the distance between CQDs decreases significantly to the point of matching, within the experimental error, the lattice parameter of PbS (*d*_100_ = 5.9 Å). The ligand removal is therefore very efficient and the NN distance is consistent with the formation of epitaxial necks.

For OA samples, the angle φ (Figure 2e) averages around 55°, suggesting that the unit cell of the SL is a distorted BCC or BCT, which is very common for ligand-capped SLs. The geometry does not change with the number of layers. Furthermore, this angle corresponds to the direction of the {101}_SL_ plane (or {011}_SL_ and symmetry-related) and is approximately half of the twinning angle previously described. It is very interesting to note that the angles (and the overall FFT symmetry) remain the same for the LE samples despite the significant reduction in lattice parameter. While it is difficult to completely determine the 3D structure of the SL from electron microscopy imaging, this finding indicates that the unit cell of the superstructure contracts rather uniformly after ligand removal maintaining a similar geometry. Therefore, the final sample preserves the {110}_SL_ orientation with respect to the substrate and most likely the unit cell becomes a distorted simple cubic (SC) or rhombohedral (Rh).

**Figure 3:**
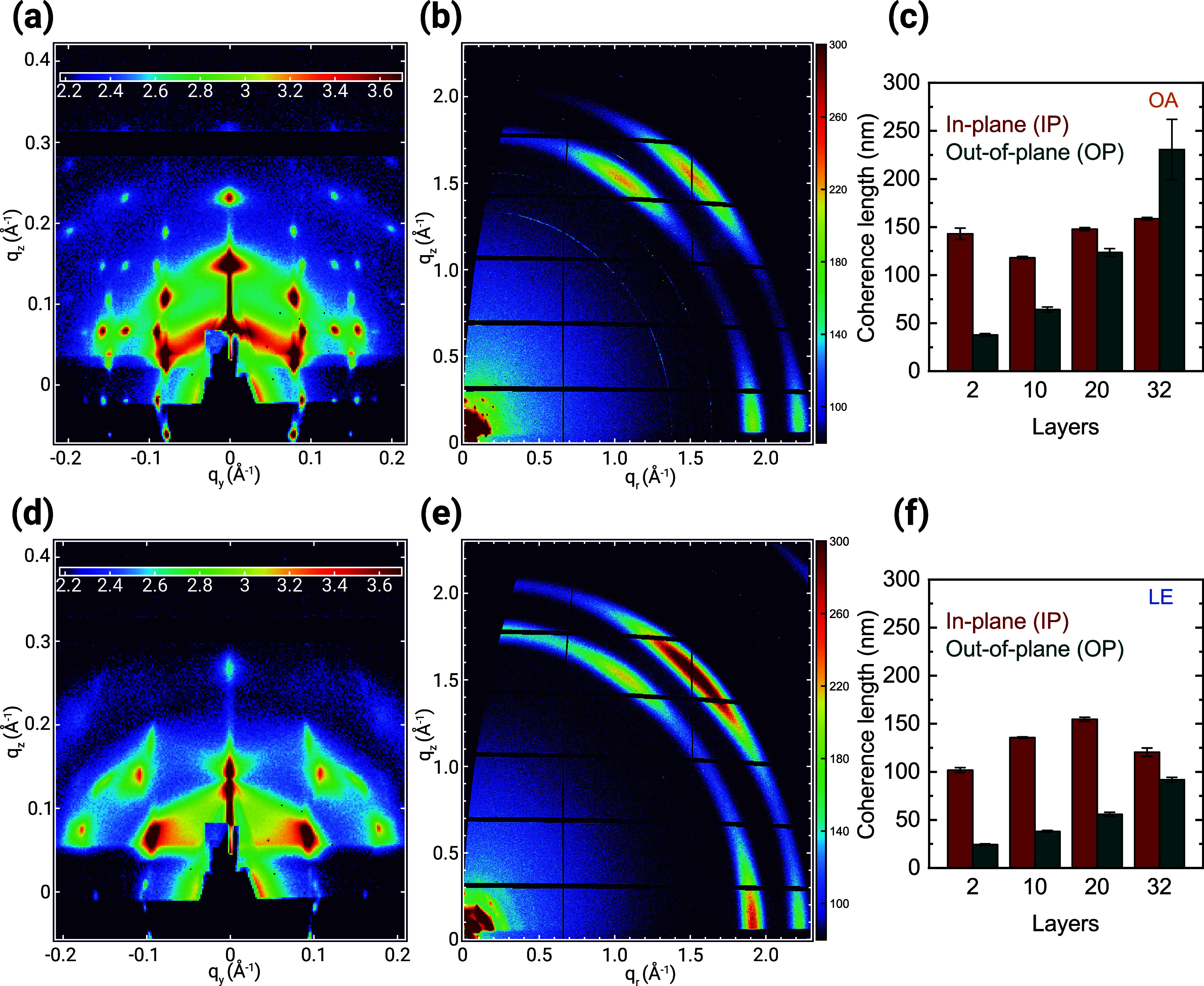
Grazing-incidence X-ray scattering measurements. (a) GISAXS pattern of the 220 nm thick OA sample. The inset scale bar is logarithmic; (b) corresponding wedge-corrected GIWAXS pattern for the sample in panel (a). The scale bar is linear for ease of visualization; (c) in-plane and out-of-plane coherence lengths for OA samples as a function of the number of layers; (d) GISAXS pattern of the 130 nm-thick LE sample. The scale bar is the same as in (a) for direct comparison; (e) corresponding wedge-corrected GIWAXS pattern for the sample in panel (d) and with the same scale bar as in panel (b); (f) IP and OP coherence lengths for the LE samples as a function of the number of layers. The *y*-axis is the same as in panel (c) for comparison.

A more extensive and large-scale structural characterization of the SLs at both the nano- and atomic scale has been performed by grazing-incidence small/wide angle X-ray scattering (GISAXS/GIWAXS), whose results are summarized in Figure 3. The GISAXS pattern of the 220 nm OA sample reveals up to three orders of sharp diffraction spots. While the width of the diffraction peaks is correlated to the domain size of the SLs, the number of reflections and their clear spot-like shape indicate a high level of ordering and low para-crystalline disorder of the sample. Using the TEM results as a starting point to index the complete set of diffraction spots observed in the GISAXS region, we could index the diffraction pattern with a single BCC unit cell (Figure S6). The structure has a slight rhombohedral distortion (α ≈ β ≈ 90°, γ ≈ 86°) and lattice parameters of *a* ≈ *b* ≈ 10.2 nm and *c* ≈ 9.8 nm. The indexing confirms that this unit cell has an orientation of 45° with respect to the substrate, meaning that the SL is aligned with the {110}_SL_ plane parallel to the substrate. Here, we have focused on the thickest sample, but these observations also hold for the rest of the samples except for the three-layer one (Figure S7).

The pattern of the thickest (130 nm) EDA-treated sample displays striking differences with respect to the OA one (Figure 3d). The number of diffraction spots has decreased, suggesting an increase of the para-crystalline disorder, but their shape remains still spot-like indicating that the three-dimensional coherency is preserved. Upon indexing the pattern, we found that the unit cell has two equal lattice vectors of 6.2 nm and one of 7.2 nm. The {110}_SL_ orientation with respect to the substrate is preserved analogously to the OA case. The rhombohedral distortion in the LE samples is strongly enhanced since α ≈ 67(2)°, β ≈ 113(3)° while γ remains constant at about 85° due to the constraints imposed by the necking (Figure S8). From simple structural considerations, the change in unit cell volume explains the thickness shrinkage after LE, which is complete throughout the structure as proven by incident angle-dependent GISAXS measurements (see Supplementary Methods, Figure S9).

In Figure 3b, we show the wedge-corrected GIWAXS pattern for the 220 nm-thick OA sample. The arc-like nature of the GIWAXS signal suggests the collective alignment of the CQDs and, consequently, of their atomic planes. By indexing the GIWAXS reflections, we evidenced that the CQDs have an overall collective atomic lattice (AL) with {110}_AL_ orientation. Nevertheless, the relatively broad angular spreading of the diffraction spots located at *q*_r_ ≈ 1.9 Å^–1^ and *q*_r_ ≈ 2.2 Å^–1^ suggests a significant deviation from a perfect collective orientation of the nanoparticles. This is nonetheless surprising since in these SLs, the CQDs are still covered with OA but already try to align in a preferential direction. This effect has already been observed in both PbS^25^ and PbSe QD SLs^46,50^ and has been explained with the different coverage of the organic ligands on the {100} and {111} facets. To allow for a quantitative comparison between samples, we took azimuthal cuts through the main diffraction spot at *q*_r_ ≈ 2.2 Å^–1^ and χ = 45° and we fitted the angle-dependent intensity with a Gaussian profile after background subtraction. The width of the Gaussian fit spans from 10.8° for the thinnest sample to 7.5° for the thickest one, suggesting that in the latter case, the collective alignment of the CQDs is improved.

The GIWAXS pattern of the LE sample (Figure 3e) shows an increased intensity of the diffraction arcs, which points to an improved collective alignment with respect to the OA case. We repeated the analysis used for the OA samples and, while we found no correlation with the different thicknesses, we observed an average decrease of the diffraction spot width of 2.1° ± 0.4° when LE was performed. This is consistent with previous observations that the epitaxial necking within the CQDs improved their collective alignment within the SLs.

To quantitatively determine the ordering of the SLs, we measured the IP and OP coherence length from the broadening of the GISAXS reflections as we showed in our recent report.^46^ For the IP coherence length, we considered a horizontal line cut along the first {110}_SL_ reflection while for the OP one, we considered a vertical line cut at *q*_*y*_ = 0. The peaks are then fitted with a pseudo-Voight function and the coherence length is calculated as 2π/fwhm (Figure 3c). For the OA SLs, the average IP coherence length is 140 ± 20 nm with no evident trend with increasing thickness, which is instead present in the vertical direction (Figure S10). Consistently with what we found for 3D PbSe CQD superlattices,^46^ the structures are fully or close to completely coherent in the OP direction, even above 200 nm. This result confirms that the 3D SLs are inherently coherent in the OP direction, *i.e.*, across the whole film’s thickness. For what concerns the EDA-treated samples (Figure 3f), in the IP direction, the average coherence length is decreased to 120 ± 30 nm but due to sample-to-sample variation the difference with the OA case is not statistically relevant (as determined by a two-sample *t*-test with 0.05 confidence level). Instead, the OP coherence length shows again a clear trend with increasing thickness. The disrupting effect of the LE is clearer in this case since for the 100 nm sample the OP coherence length is only 56 nm and the 130 nm one retains 92 nm. We believe that this is still an excellent result, and the structural coherency of 3D SLs could have important implications in technological applications where the standard device architecture is a stack and charge transport occurs in the OP direction.

**Figure 4:**
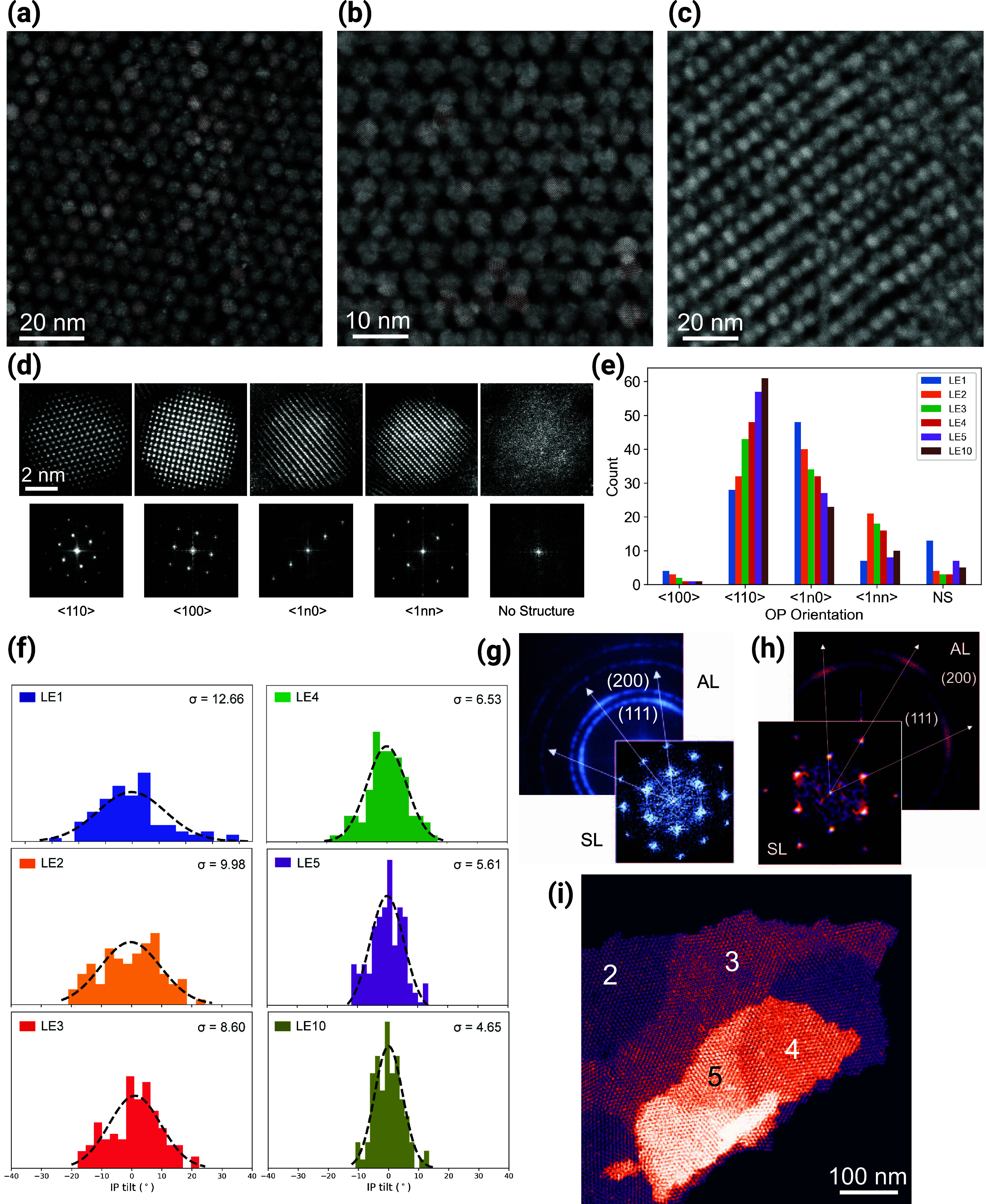
High-resolution STEM-HAADF images of (a) mono-, (b) bi-, and (c) ten-layer superlattices after LE. (d) STEM-HAADF images of CQDs in different OP orientations along with the respective FFTs (NS: No structure observable). (e) Population distribution of CQDs with their OP orientations for samples with 1, 2, 3, 4, 5, and 10 layers after LE. (f) Population distribution of CQDs with their IP tilts with respect to one of the SL axes for the same samples in panel (e). The standard deviation (σ) in all the plots is calculated assuming the Gaussian distribution of the IP tilt. (g, h) FFTs of AL and SL for samples with 2 and 10 layers after LE. The arrows indicate the AL and SL alignment. (i) STEM-HAADF image of the area of SL with multiple layers. Numbers indicate the count of layers.

High-resolution (HR) STEM-HAADF imaging was performed to get insights into the correlation between SL and atomic orientations in the in-plane (IP) direction. Images are shown in Figure 4a–c for samples with 1, 2, and 10 layers after ligand exchange (LE). With this technique, it is possible to achieve atomic resolution while imaging large amounts of CQDs in a single-crystalline domain. These high-resolution images also contain information on the orientation distribution of CQDs in the out-of-plane (OP) and in-plane (IP) directions. The HR images of the CQDs oriented in different OP directions are shown in Figure 4d along with the respective FFTs. This includes CQDs oriented in {100}_AL_ and {110}_AL_ directions, which are clearly visible in high-resolution images for all the samples. The CQDs oriented in the directions (1n0)_AL_ and (1nn)_AL_, which refer to their OP tilt toward the (110)_AL_ and (111)_AL_ directions, respectively, are observed in all the samples in large amounts. A significant fraction of the CQDs is tilted in these directions due to the disorder in the sample. The mono and bilayered superlattices show a small fraction of {110}_AL_ OP orientations of the CQDs (Figure 4e). The SL with five and ten layers shows the domination of the {110}_AL_ OP orientations of the CQDs over a very small fraction of other orientations. From these high-resolution images, information on the IP tilts of the CQDs from the high symmetry axis of SL is extracted (Figure 4f). A comparison of the samples with increasing thickness suggests a significant reduction in the tilt range from ±35° to ±15° along with a decrease in the standard deviation for the IP tilts from 12.6° to 4.6°. This confirms the high degree of coherence in all the directions along with the higher ordering in the thick SLs. The FFTs extracted from two and ten-layered samples show the AL and SL alignment with {110}_SL_ ∥ {110}_AL_ and {200}_SL_ ∥ {200}_AL_ (Figure 4g,h, respectively). This suggests a good alignment of AL and SL in both thin and thick samples with a strong difference in the reduction of the IP tilts spreads in thick samples as observable in the FFT in Figure 4h, which supports the better ordering in thick samples. Figure 4i shows the SLs with different layers from 2 to 5.

After the in-depth structural characterization, we studied the transport properties of the fabricated SLs employing ion gel-gated field-effect transistors (IGFET). This technique relies on the use of the electrical double layer (EDL) to accumulate a high charge carrier density in the film (above 10^14^ cm^–2^ in optimal conditions).^9,52−54^ In this way, allowing to fill a considerable amount of trap states in the CQD solid, it is possible to measure the close-to intrinsic transport properties of the SLs.^8,55^ The problem of trap density is highly relevant especially for lead chalcogenide CQD SLs as evidenced by the moderate mobilities that can be achieved in solid-state gated transistors.^20,29,50^ In recent years, the use of EDL to gate SL transistors has enabled the understanding of the full potential of both PbS^39,41^ and PbSe assemblies.^22,43,46^ As mentioned earlier, in a recent work, we demonstrated electron mobilities up to 270 cm^2^ V^–1^ s^–1^ in three-dimensional PbSe CQD superlattices, owing to the much-improved ordering of these structures with respect to the 2D ones.^46^ Encouraged by this result, we set out to study the transport properties of PbS superlattices, which show high coherence lengths in both IP and OP directions.

The gating was done through an ion gel of [EMIM][TFSI] in a P(VDF-HFP) copolymeric matrix.^22,46^ We dedicated specific attention to the characterization of EDL by comparing the capacitances obtained with two different methods based on impedance spectroscopy (see Supplementary Methods). In particular, the capacitance was measured on the actual IGFETs and as a function of frequency and gate voltage (Figure S11). To consider the electrode’s geometry, we used a platinum wire inserted into the bulk of the ion gel to accurately measure the potential drop at the interface with the SL. The device had a channel width of 20 μm and variable channel length between 1 and 13 μm. Using devices of such small dimensions ensured characterizing transport across the least number of grains possible, thus minimizing the detrimental effect of structural defects of the superlattices.

**Figure 5:**
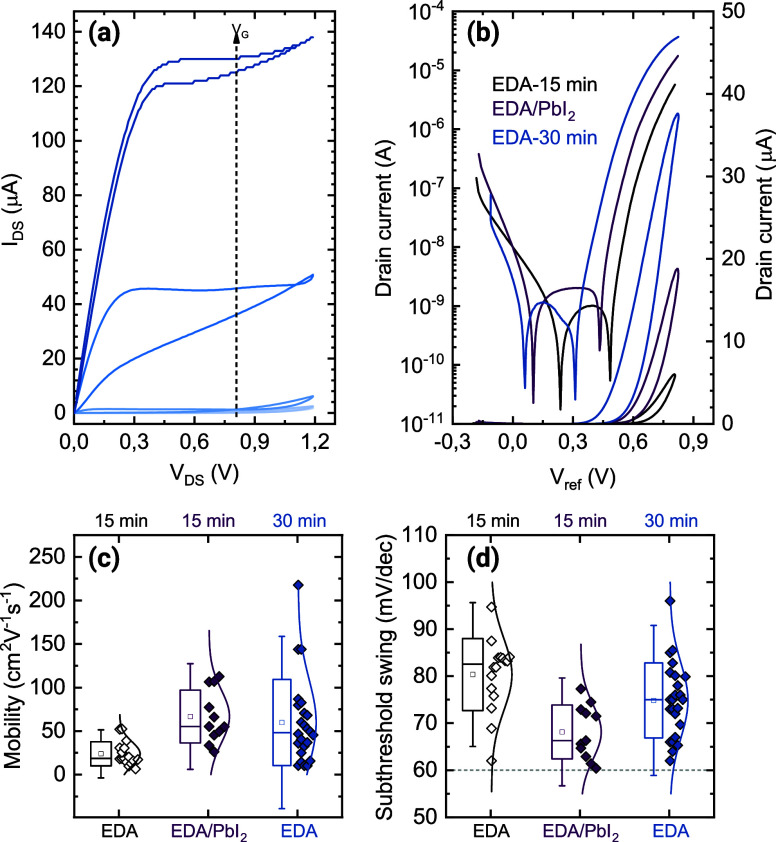
Transport properties of the superlattices. (a) Output curve of the best-performing device made 52 nm thick, EDA-treated for 30 min superlattice. The gate voltages are changed from 0.3 to 1.9 V in steps of 400 mV; (b) transfer curves of the best devices for each condition in both logarithmic (left axis) and linear scale (right axis). For the curves in a linear scale, the reverse sweep is shown to compare the hysteresis; (c) comparison of measured mobilities and (d) subthreshold swings for the three different treatments used in this work. The bottom axis specifies whether only EDA was used or if a PbI_2_ passivation was implemented. The top axis specifies the duration of ligand exchange with EDA. The gray dashed line indicates the Boltzmann limit for the subthreshold swing.

Figure 5a shows the output curve of the best-performing device with a thickness of 52 nm, obtained with a 30 min EDA treatment. For several reasons concerning the working mechanism of the chosen ionic liquid, we focus solely on electron transport.^22,41^ As expected from the off-stoichiometric composition of the CQDs, the IGFETs show clear n-type behavior,^56^ with currents of tenths of microamperes in the linear regime and over 100 μA in the saturation regime. The curves show high modulation and moderate to low hysteresis, suggesting that trapping mechanisms might still be present despite the high charge accumulation.

The corresponding transfer curve in the linear regime (*V*_DS_ = 100 mV) shows several interesting features (Figure 5b). The transport is confirmed to be strongly n-type and with a clear off-region and high on/off ratio of 10^5^–10^6^. Together with the retainment of the excitonic peak, such high modulation is a strong sign that quantum confinement is preserved and that the CQDs are not sintered during the annealing step nor with the longer LE. The gate leakage current is minimal and always less than 10 nA, indicating that the transistor is correctly operated in the electro-static regime. From the transfer curves in linear scale, we can confirm a moderate counterclockwise hysteresis, which is due to the slow ion motion at the interface with the SL. The threshold voltage is quite high (≈0.6 V) and above it, the curves do not show any sign of band-filling. All the observations made so far hold for the entire set of measured devices fabricated in the three different conditions (more than 50), especially for what concerns the polarity (n-type), the “on” currents (1–10 μA), the on/off ratio (10^4^ - 10^6^) and the low leakage. Nevertheless, we found interesting differences in the samples, depending on the duration of the ligand exchange process and the post-treatment, affecting mostly the mobility, subthreshold swing (SS), and threshold voltage.

The 30 min LE sample achieved maximum mobility of 222 cm^2^ V^–1^ s^–1^ (Figure 5c) with an average of 60 ± 50 cm^2^ V^–1^ s^–1^ but showed significant variation among devices and depending on the thickness of the film (Figure S12). The very long LE allows the removal of almost the totality of OA, but it negatively affects the adhesion of the film on the substrate leading to delamination and severe cracking of the flakes upon deposition (as mentioned in the discussion over the FTIR spectra). The SS of these samples is 75 ± 8 mV/dec (Figure 5d), rather far from the ideal Boltzmann limit of 60 mV/dec that is expected for trap-free FETs and that has been observed in PbS QD 2D SLs.^41^ This indicates an excessive surface trap density that can only be partially filled with the ion gel and is most probably derived by the aggressive EDA ligand removal that leaves the CQDs facets unterminated.^5^

Reducing the LE time to 15 min dramatically reduces the average mobility to 24 ± 14 cm^2^ V^–1^ s^–1^, while further increasing the SS to 80 ± 8 mV/dec. This indicates that a significant number of organic ligands is left on the surface of the CQDs hampering charge transport. These samples also display the highest threshold voltages (Figure S13). Nevertheless, these samples showed improved adherence to the substrates and a narrower mobility distribution. We hypothesized that by further removing OA and passivating the CQD surface traps these samples could be largely improved. Halogen-based ligands are the most widely used for electronic applications and have been proven to effectively passivate traps mostly by terminating the lead-rich {111} facets of the Pb-chalcogenide CQDs.^57^ Furthermore, these ligands do not alter the optical and electronic properties of the nanocrystals and the n-type behavior is either preserved or enhanced. We therefore chose to employ the prototypical PbI_2_ ligand. Besides the halide passivation, it has been proposed that lead ions can furthermore repair the surface of the CQDs since some atoms are stripped away as lead-oleate compounds.^58^ This is extremely relevant, as the epitaxial necking is due to atomic diffusion from the {111} facets to the {100} ones.^50^

Indeed, implementing PbI_2_ passivation after 15 min of EDA treatment results in average mobilities 67 ± 30 cm^2^ V^–1^ s^–1^, bridging the gap with the 30 min treated samples and narrowing the mobility distribution. More interestingly, the passivation significantly reduces the SS to 68 ± 6 mV/dec with several devices displaying values close to the theoretical Boltzmann limit (Figure 5d). The statistical significance of this difference is once again proven with a *t*-test, indicating that the two distributions (PbI_2_-passivated and 30 min LE) are significantly different (α = 0.009). Thus, we can conclude that lead iodide is very efficient in passivating the unterminated facets of the CQDs superlattices, therefore significantly reducing the surface trap density. This finding is very important as it demonstrates that CQD superlattices can be passivated after assembly, giving extra flexibility in their fabrication.

The threshold voltage also shows a clear trend with the three different treatments, namely, 0.74 ± 0.04 V for 15 min EDA, 0.63 ± 0.07 V for 15 min EDA/PbI_2_, and 0.51 ± 0.14 V for 30 min EDA (Figure S13). We observe a significant broadening in the distribution for the 30 min EDA LE, meaning that this treatment is increasing the energetic disorder in the sample. The 15 min samples instead show narrower distributions and we interpret the decrease in threshold voltage as a modification of the CQD surface due to the iodide passivation in the treated sample.^59^

We conclude with some comments on the transport properties so far discussed and in the context of the current understanding of this class of systems. Recent work on 2D PbS SLs has claimed to have reached the boundary of the metallic-to-insulator transition as proven by temperature-independent conductance at high carrier density (≥10^14^ cm^–2^) despite still moderate mobility (15 cm^2^ V^–1^ s^–1^).^41^ In our work instead, we have demonstrated electron mobilities above 200 cm^2^ V^–1^ s^–1^ for PbS-based 3D systems at lower carrier density (∼2 × 10^13^ cm^–2^ at 2 V, Figure S11d). Even below band-filling conditions, several of the devices presented in this work show high conductivity, and the actual values are likely underestimated since the contact resistance was not considered in our measurements. This result is of great importance since achieving high mobility without band-filling ensures avoidance of bleaching of the excitonic peak in absorption, a well-known phenomenon that was demonstrated in electrolyte-gated transistors at high accumulated carrier density.^60^

Historically, PbSe CQDs have been preferred for the formation of SLs since their bulk counterparts show higher mobilities with respect to PbS (1000 cm^2^ V^–1^ s^–1^ against 600 cm^2^ V^–1^ s^–1^).^6,61^ However, there is insufficient theoretical knowledge to understand whether this difference should be encountered also in superlattices of the quantum-confined counterparts. In our experiments, we observed significantly better mobilities for PbSe with respect to PbS. We notice though that the SLs that we could obtain with the two systems present a crucial difference: the collective orientational alignment of the CQDs is significantly better for PbSe rather than PbS, showing clear diffraction spots in the GIWAXS pattern. In contrast, the samples in this work show rather arc-like shape diffraction features. The same difference can be observed also in high-resolution STEM-HAADF imaging, where the misalignment between CQD results in elongated reflections in the FFTs of the images (Figure 4g,h). This is a crucial difference since it affects the neck formation and growth and the connectivity of the SL. These parameters have been proven to be fundamental for achieving charge delocalization.^45^ Furthermore, computational studies have shown that the highest degree of delocalization and therefore mobility in 3D SLs of PbS CQDs is achieved with the simple cubic structures owing to a maximized wave function overlap between neighboring {100} facets.^15^

Nevertheless, in both cases mobility values above 200 cm^2^ V^–1^ s^–1^ could be obtained. It is also clear that samples in this work have an improved translational ordering and the coherence lengths are significantly higher than in our previous work on PbSe SLs. Altogether with the larger grains and absence of pinholes, this could make up for the worse orientational alignment and still enable us to achieve very high mobilities. The observed dissimilarities among samples with different treatments and LE highlight once more that trap passivation at the CQD surface is the most important challenge to overcome. This problem has hardly been studied in SLs, but it will be necessary to address this issue in the future to fully exploit the potential of these metamaterials for technological applications.

## Conclusions

In this work, we have demonstrated the fabrication of 3D superlattices by self-assembly of highly monodisperse PbS CQDs. With our method, we could grow SLs with control over thickness up to 220 nm and with excellent coverage and large grain size. We implemented an EDA treatment that enabled the full removal of ligands throughout such thick samples while retaining the optical properties of the CQDs. Advanced structural characterization techniques revealed that the samples have a high degree of translational order with significant in-plane and out-of-plane coherence lengths, even after ligand exchange. Ion-gel gated field-effect transistors were utilized to investigate the transport of the superlattices, which had different ligand exchange times. Electron mobilities up to 220 cm^2^ V^–1^ s^–1^ were recorded, on par with analogous results obtained with PbSe CQDs despite the less pronounced truncation of the PbS ones and the consequently increased orientational disorder. Interestingly, devices fabricated with shorter EDA exchange time showed high subthreshold swings despite using the ion gel as the gate dielectric, pointing to a very high density of traps. A postdeposition passivation treatment with PbI_2_ largely decreased the subthreshold swings and therefore the trap density. Altogether, our findings propose a way to fabricate thick, electronically coupled SLs of PbS CQDs. The high absorption in the short-wavelength infrared combined with the very high mobility and the superior stability of the PbS make these metamaterials ideal candidates for the next generation of optoelectronic devices to detect light. These results furthermore indicate that trap density will be the fundamental limitation to overcome for enabling the technological application of these systems.

## Materials and Methods

### Materials

PbS CQDs were obtained from Quantum Solutions. Octane (99+%, extra dry, Fisher), ethylene glycol (99.8%, anhydrous, Fisher), acetonitrile (99.9+%, extra dry, fisher), ethylenediamine (99+%, anhydrous, Fisher), lead(II) iodide (99.99%, TCI), dimethyl sulfoxide (99.9%, anhydrous, Sigma), poly(vinylidene fluoride-*co*-hexafluoropropylene) (P(VDF-HFP), Sigma), 1-ethyl-3-methylimidazolium bis(trifluoromethyl sulfonyl)imide [EMIM][TFSI] (≥98%, Sigma), and cyclohexanone (99.8%, Fisher). All materials were used as is and were opened and stored always in nitrogen-filled gloveboxes.

### Superlattice Formation

Teflon baths with size 1.5× 1.5 × 1.5 cm^3^ were first rinsed with octane, then sonicated in acetone and isopropanol, and blow-dried with a nitrogen gun. For all the experiments, the Teflon bath was filled with 2 mL of EG. The CQD solution was prepared by dissolving 6.2 nm PbS nanocrystals in octane at a concentration of 65 μM (≈41 mg/mL) and then filtered with a 0.2 μm PTFE filter. To form the superlattices, the CQD solution was drop-casted at the center of the Teflon bath using a micropipette. To tune the thickness in the above-mentioned range, volumes between 0.33 and 3.27 μL were used. The solvent evaporation and CQD self-assembly lasted 10 min after which the samples were either collected or LE was performed. In the latter case, a solution of 5 M EDA in EG (100 μL) was injected into the subphase with a syringe and left reacting for either 15 or 30 min. The samples were collected on a variety of substrates and immediately immersed in pure acetonitrile for 30 s to remove excess EG, EDA, and OA. When performed, the extra passivation step was achieved by immersing the substrates first in a 10 mM PbI_2_ solution in DMSO for 5 min and then in a pure DMSO solution to remove unreacted lead iodide. Eventual annealing was performed on a hot plate at 120 °C for 20 min. All samples were dried overnight in mbar vacuum. All the processes were carried out in a nitrogen-filled glovebox (<0.1 ppm of O_2_/H_2_O).

### Ion-Gel Preparation

The solution was prepared as in our previous work.^46^ P(VDF-HFP), [EMIM][TFSI], and cyclohexanone were mixed in a 1:4:7 ratio and stirred at 70 °C and 1000 rpm overnight. Before every deposition, the solution was again warmed up to 70 °C and stirred for 1 h to ensure homogeneity. All the IGFETs were covered with a droplet of ion-gel (0.3 μL) then annealed at 70 °C for 1 h and dried overnight in a mbar vacuum.

### Absorption Measurements

The absorption spectra of the CQD solution and the superlattice films were carried out on a Shimadzu UV3600 spectrometer. For solutions, optical glass cuvettes were used, and the spectra were background corrected for the pure solvent (octane). The concentration and size of the PbS CQDs were determined from a well-established procedure in the literature.^4^ For thin films, the superlattices were deposited on optical glass that was sonicated in water, acetone, and isopropanol and baked in an oven for 1 h at 100 °C. A neat glass substrate was used to correct the background.

### FTIR

The spectra were acquired with a Shimadzu IRTracer-100 in transmission mode with 60 scans. The superlattices were deposited onto double-side polished Si/SiO_2_ (525 μm, native oxide). The substrates were cleaned with sonication in acetone and isopropanol and dried in the oven.

### Atomic Force Microscopy (AFM)

Micrographs of the superlattices were collected with a Bruker MultiMode-8 microscope equipped with ScanAsyst automatic image optimization. Gwyddion data analysis software was used to analyze the images and extract the thickness of the samples.

### Electron Microscopy

Low-magnification STEM-HAADF and SE imaging was performed with an FEI Helios G4 CX operated at 30 kV. Atomic-resolution STEM-HAADF imaging was performed using a monochromated and probe-corrected Thermo Fisher Scientific Themis Z S/TEM system operated at 300 kV with beam convergence of 24 mrad. All the samples were collected onto 3 nm carbon-coated TEM grids with 400 mesh.

### Grazing-Incidence X-ray Scattering

GISAXS and GIWAXS measurements have been performed at the I07 beamline, in DIAMOND, UK. The beamline is equipped with a Huber 2 + 3 diffractometer integrating a hexapod positioning system (Breva, Symmetrie) for sample positioning and alignment. The beamline is also equipped with a 3-axis positioning system for a large area detector for GISAXS/GIWAXS experiments. The samples were measured at given incident angles using a Pilatus 2 M detector placed 575 mm away from the sample. The X-ray energy of the beam was set to 12 keV and the angular range was calibrated using the diffraction rings from standard silver behenate and silicon powder samples. The patterns were further analyzed using the GIXSGUI software in MATLAB.^62^

### IGFET Characterization

Custom patterned FET substrates were fabricated via UV photolithography following our previously reported procedure.^46^ The devices were measured using an Agilent E5270B semiconductor parameter analyzer in a nitrogen-filled glovebox (<0.1 ppm of O_2_/H_2_O). The gate voltage was applied through a platinum foil gently laid on the ion-gel droplet while a platinum wire inserted into the droplet measured the potential difference between the bulk of the electrolyte and the superlattices. The transfer curves were acquired at a drain-source bias of 100 mV. The gate voltage was swept at a rate of 5.7 mV/s. Linear mobilities were calculated with the formula:
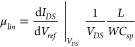
1

### Impedance Spectroscopy

The specific capacitance of the devices was measured with a Solartron 1260 impedance analyzer. The frequency sweeps were performed in a range of 10^–2^–10^6^ Hz. The gate voltage sweeps were performed in the range of −0.5 to 2 V with an AC voltage of 50 mV and a frequency of 570 mHz (see Supplementary Methods for details).

## Supporting Information Available

Absorption spectra, FTIR spectra, AFM, supplementary electron microscopy and X-ray scattering data and analysis, impedance spectroscopy methods and data analysis, and additional transport measurements (PDF)
