# Supplementary material for: PbI2 Passivation of Three Dimensional PbS Quantum Dot Superlattices Toward Optoelectronic Metamaterials
Source: ACS Nano. 2024 Jul 2;18(29):19124–36. doi: 10.1021/acsnano.4c04076 (PMC11271184; doi:10.1021/acsnano.4c04076)
Supplement: Supplementary file 1 — nn4c04076_si_001.pdf [file nn4c04076_si_001.pdf]

Supplementary information for

PbI_2 Passivation Of 3D PbS Quantum Dot Superlattices Towards Optoelectronic Metamaterials

Jacopo Pinna[a], Elisa Pili[a], Razieh Mehrabi Koushki[a], Dnyaneshwar Gavhane[a], Francesco Carlà[b], Bart J. Kooi[a], Giuseppe Portale[a], Maria Antonietta Loi[a*]

[a]Zernike Institute for Advanced Materials, University of Groningen; Nijenborgh 4, 9747 AG Groningen, The Netherlands.

[b]Diamond Light Source Ltd, Diamond House, Harwell Science and Innovation Campus, Didcot, Oxfordshire, OX11 0DE, United Kingdom.

*Corresponding author: m.a.loi@rug.nl

This file includes:

Figures S1 to S13

Supplementary Methods

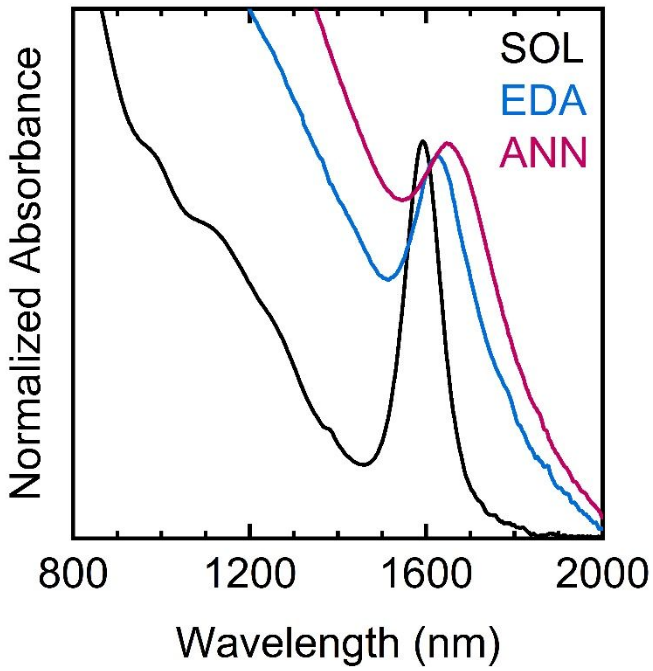

Figure S1. Normalized absorption spectra of CQDs solution (SOL), a 130 nm thick EDA-treated for 30 minutes sample (EDA), and the same sample after 20 minutes of annealing 120 °C (ANN).

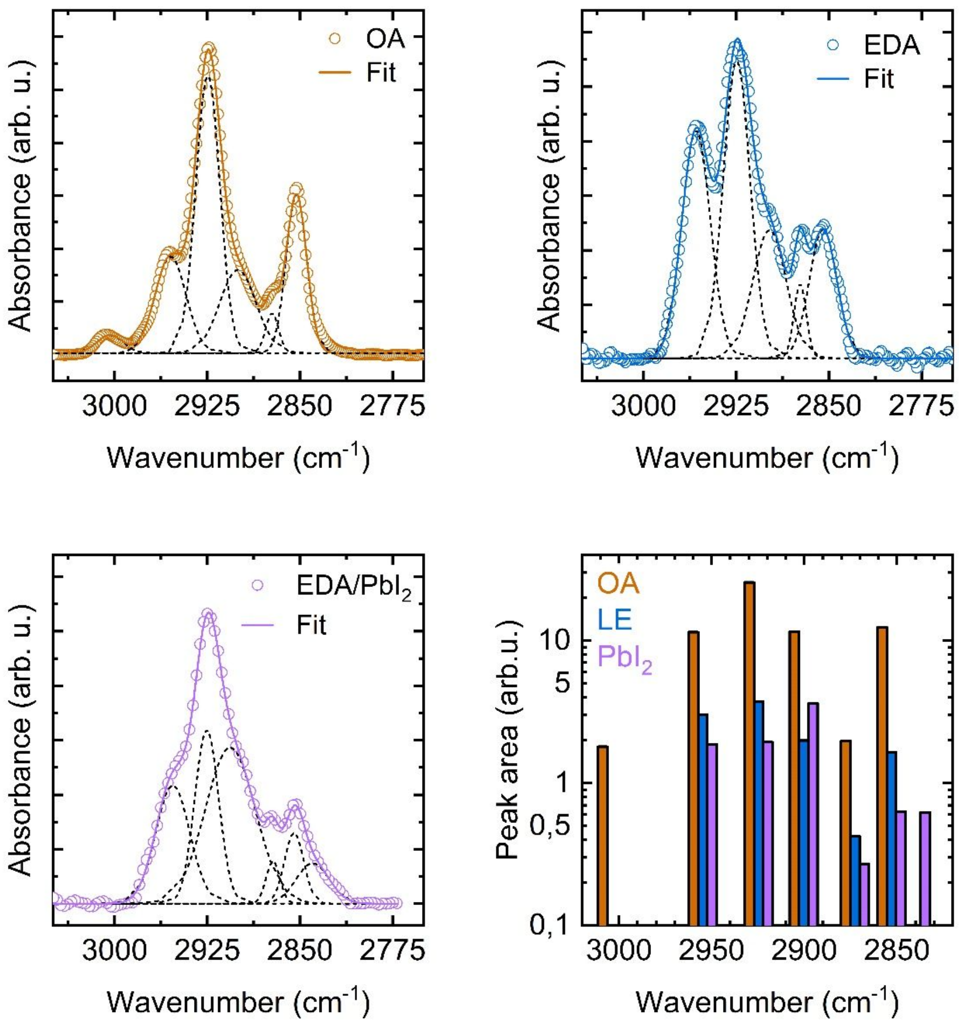

Figure S2. Full profile fitting of the FTIR spectra in the C-H vibrational region (3010-2840 cm^{-1}) for the three samples described in the main text. The fitting was performed after background correction and normalization of the spectra for the excitonic peak. For comparison, The areas of the corresponding peaks for each sample are plotted as a bar plot. The peak at 3006 cm^{-1} is present only in the OA sample while the one at 2840 cm^{-1} is present only in the PbI_2 sample.

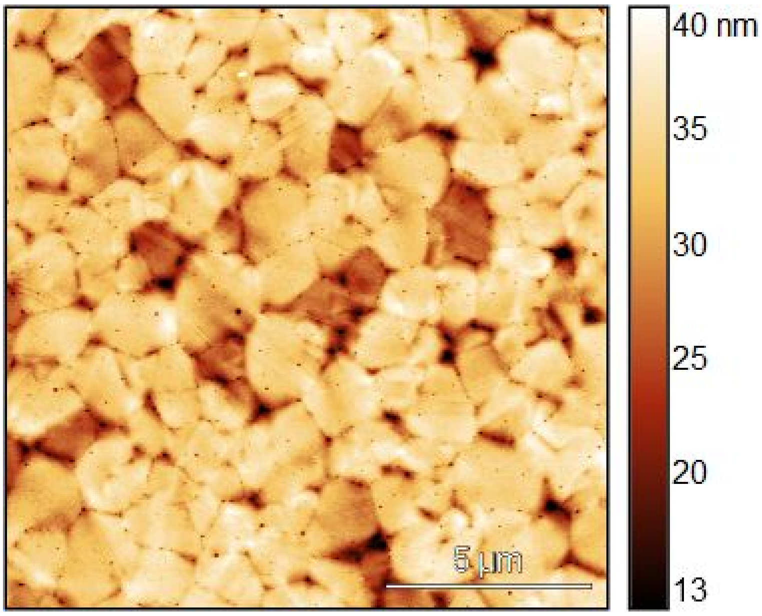

Figure S3. AFM micrograph of the 32-layer thick OA sample showing the thickness homogeneity and the large grain size.

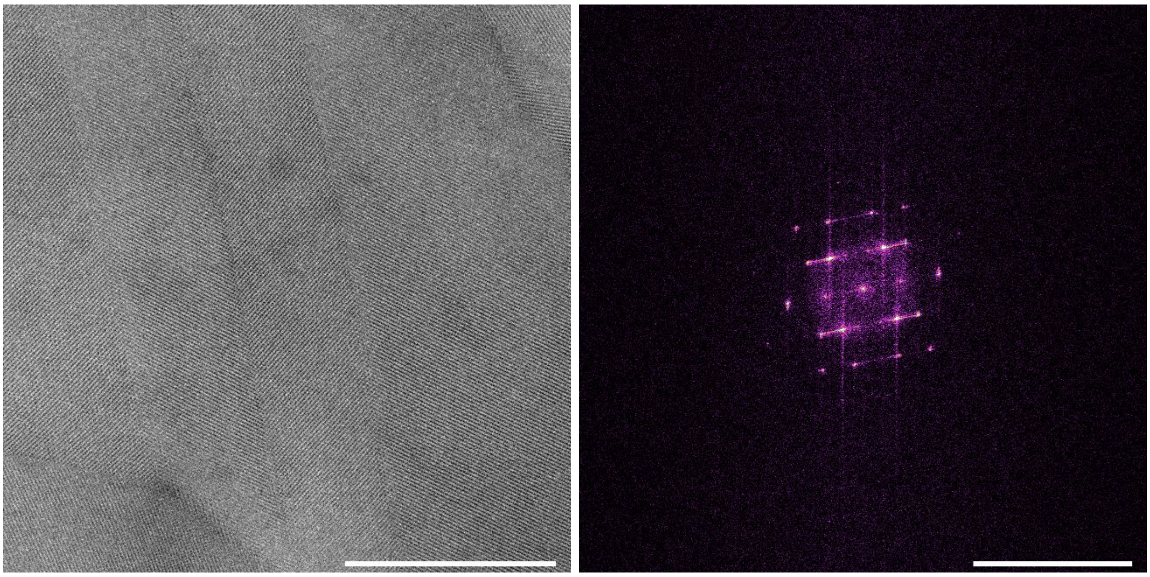

Figure S4. STEM-HAADF micrograph (left) and corresponding FFT (right) showing a region with several twinned domains. The FFT displays two identical patterns at a specific twisting angle of 108°. These twinned domains were observed in the thicker OA samples. (Scale bars: 400 nm, FFT: 0.5 nm^{-1})

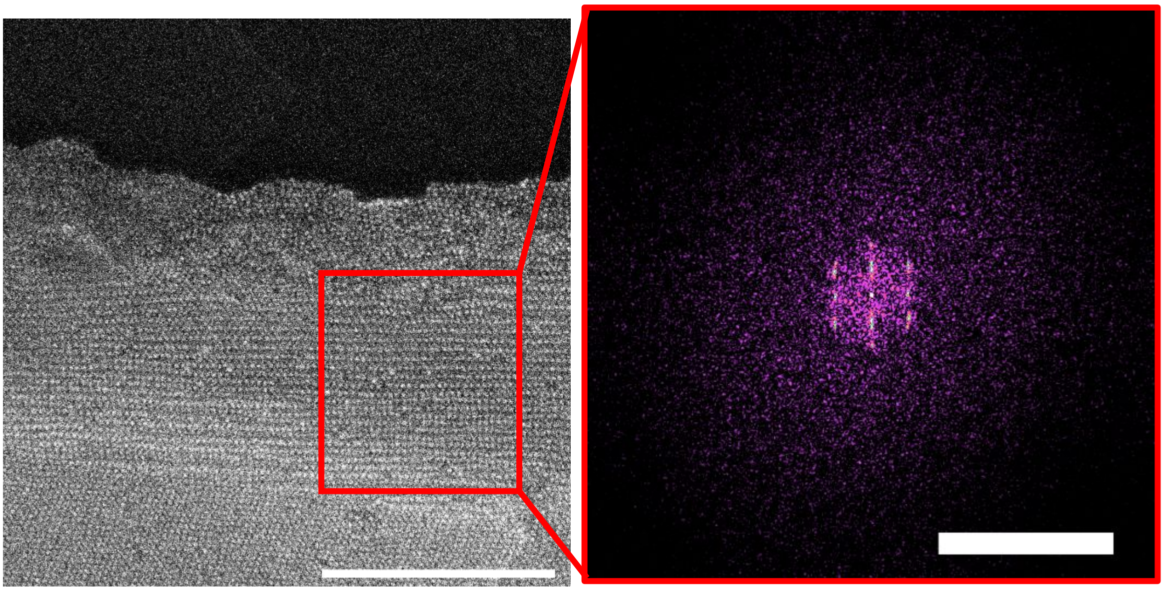

Figure S5. SE micrograph (crop of Figure 2b in the main text) showing a superlattice grain's edge in a macroscopic crack. The ordered layered structure of the CQDs is confirmed by the FFT in the highlighted region of the edge. (Scale bars: 300 nm, FFT: 0.5 nm^{-1})

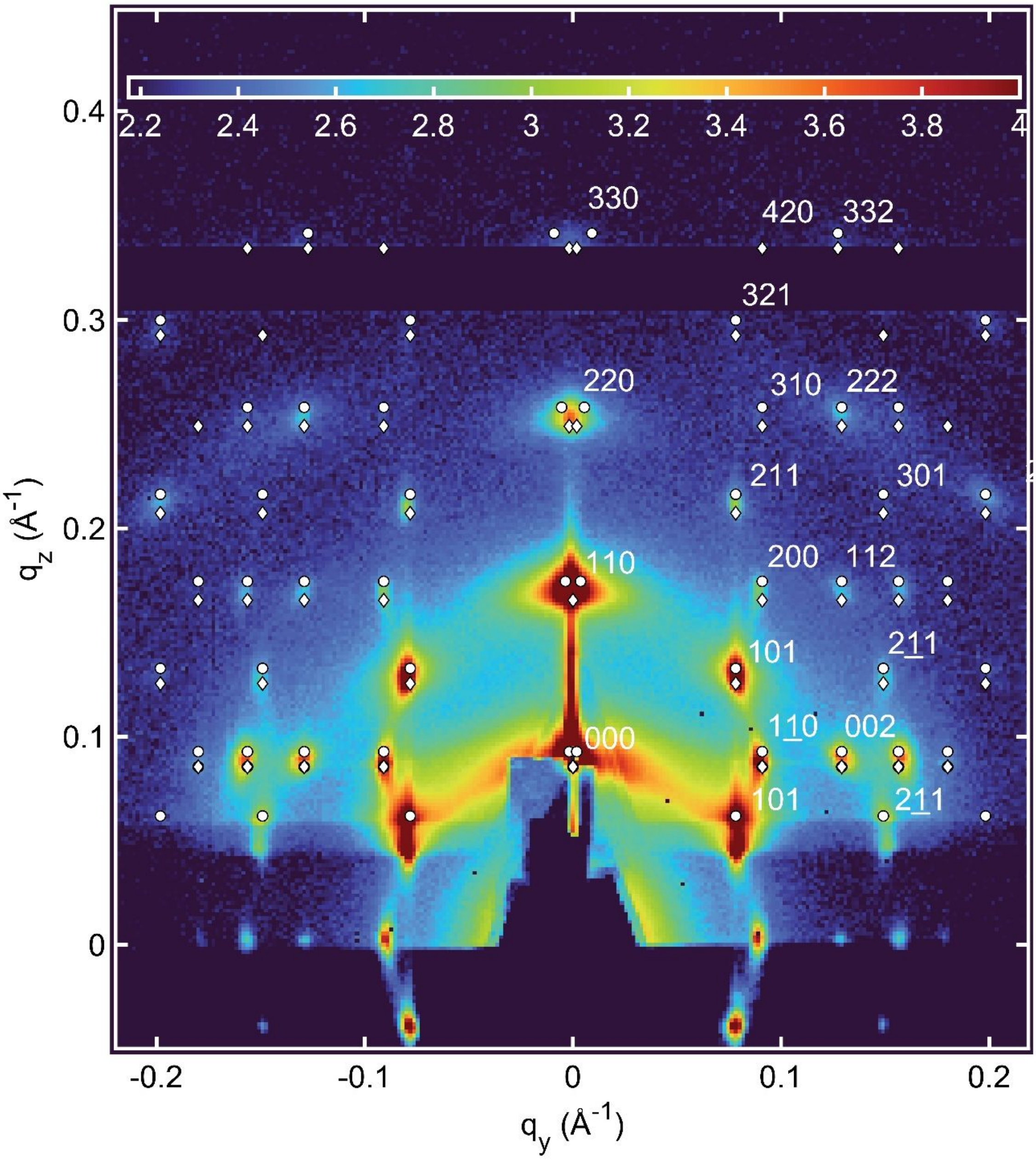

Figure S6. Indexed GISAXS pattern of the 32-layer oleic acid sample. The white rhombuses (circles) indicate the calculated reflections for the reflected (refracted) beam. The BCC unit cell is indicated in the main text. For clarity, only some of the labels are shown at the top-right of the corresponding reflection. The intensity scale is logarithmic.

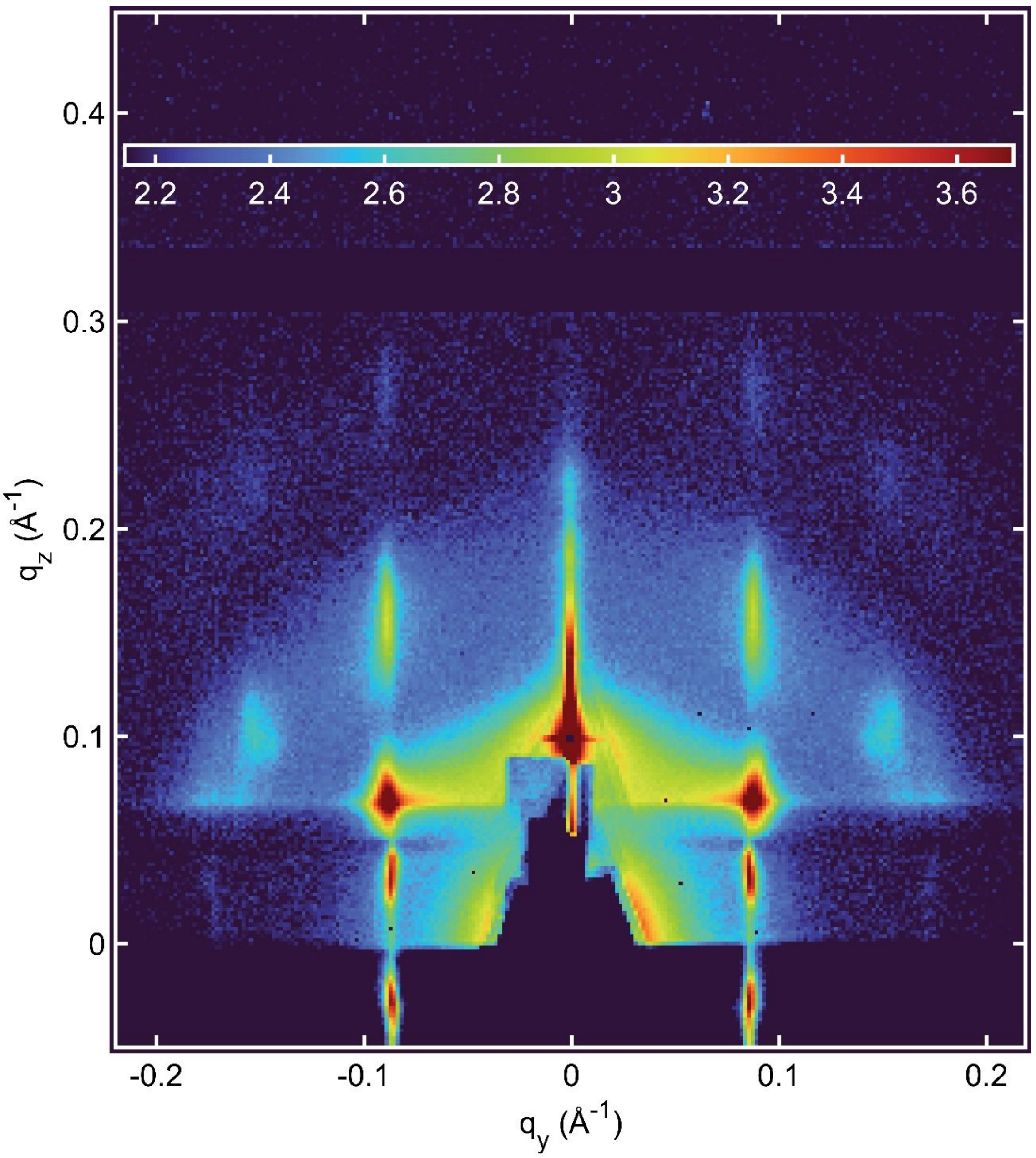

Figure S7. GISAXS pattern of the 3-layer oleic acid sample. The intensity scale is logarithmic. In this thin sample, a mixture of one, two, and three layers has been observed, with either hexagonal ($\{001\}_{SL}$ HCP) or $\{111\}_{SL}$ FCC unit cell. The GISAXS pattern shows a significantly reduced number of reflections (with respect to the thicker samples) which have an elongated, rod-like shape in the q_z direction. Instead, the first in-plane (IP) reflection is spot-like and narrow, indicating good in-plane ordering and well-defined periodicity. Given the two-dimensional nature of the scattering pattern, indexing with a 3D unit cell is not performed.

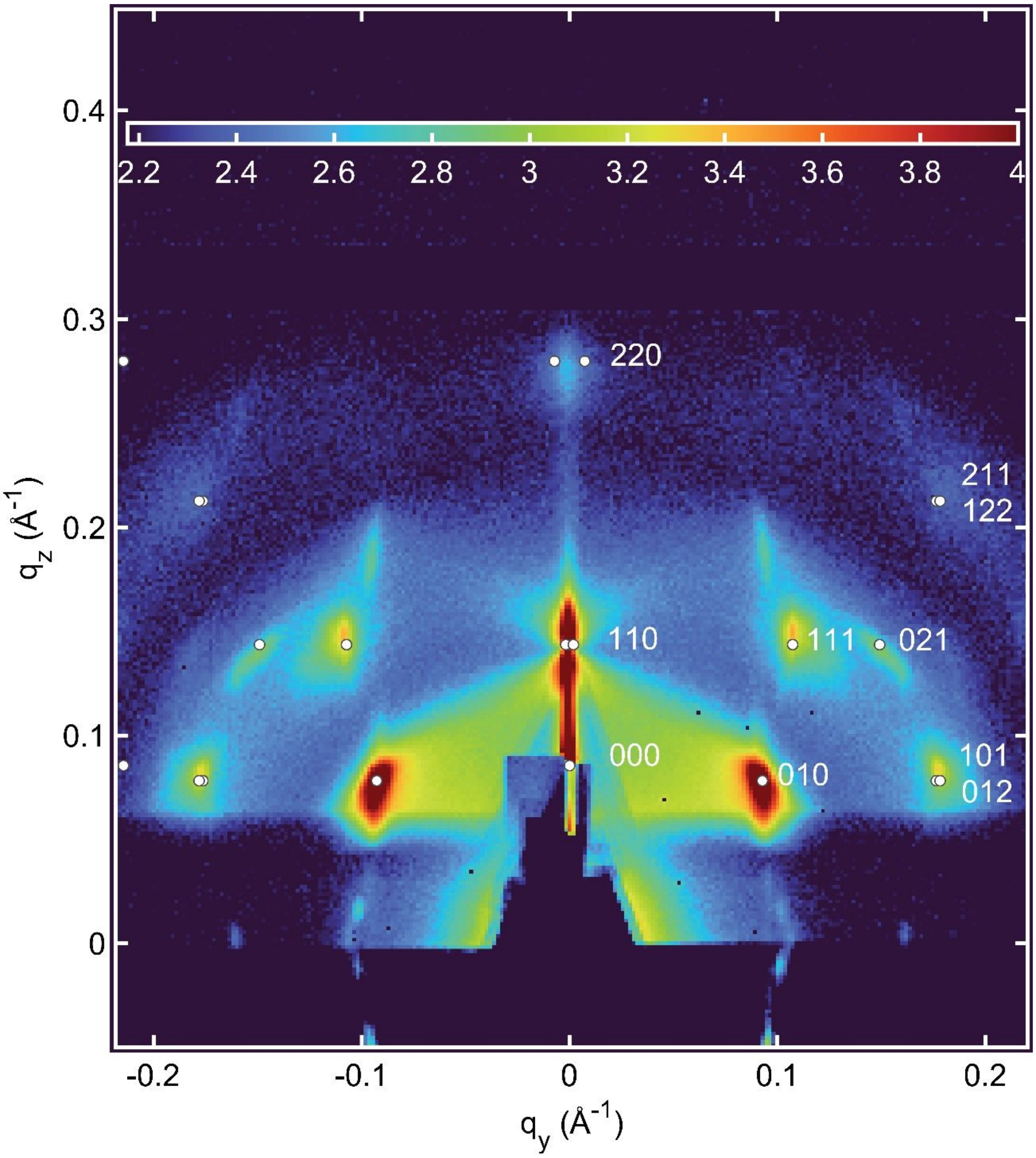

Figure S8. Indexed GISAXS pattern of the 32-layer ligand exchange sample. The white circles indicate the calculated reflections for the refracted beam. The transmitted reflections are not observed due to the compactness and density of the sample and considering that the incident angle (α_i=0.4°) is at least double the critical angle. The pattern is indexed to a distorted pseudo-rhombohedral unit cell with orientation $\{110\}_{SL}$ with respect to the substrate (a=b=6.2 nm, c=7.2 nm; α=67°, β=113°, γ=85°). The streak-like reflections are due to minor impurities in the sample. The intensity scale is logarithmic.

Supplementary Methods:

Thickness shrinkage after ligand exchange

With the determination of the SL unit cells in the OA and EDA-treated samples, we can further comment on the thickness shrinkage after LE. The $\{110\}_{SL}$ BCC unit cell has a lattice parameter of around 10 nm so, considering its orientation with respect to the substrate, the distance between consecutive layers in the vertical direction is 7 nm. For the thickest sample with a measured thickness of 220 nm, 32 layers of CQDs are present. After LE, the lattice parameter shrinks to 6.2 nm, and while the orientation of the unit cell is still the $\{110\}_{SL}$, the increased rhombohedral distortion decreases the layer-to-layer distance to 4.2 nm (the layers are now partially overlapped). If we consider that the number of layers remains the same (32), we then get a thickness of 134 nm in agreement with the one measured by AFM. For the 10 and 20 layers the effect is comparable, within experimental deviations from sample to sample. We can thus conclude that the change in thickness depends solely on the ligand removal and unit cell transformation, and no CQDs are lost in the subphase during the LE process. Interestingly, the LE is complete through the whole structure despite the significant thickness as confirmed by incident angle-dependent GISAXS patterns which show the unchanged structure and lattice parameters when probing occurs from more superficial to deeper regions of the sample (Figure S9).

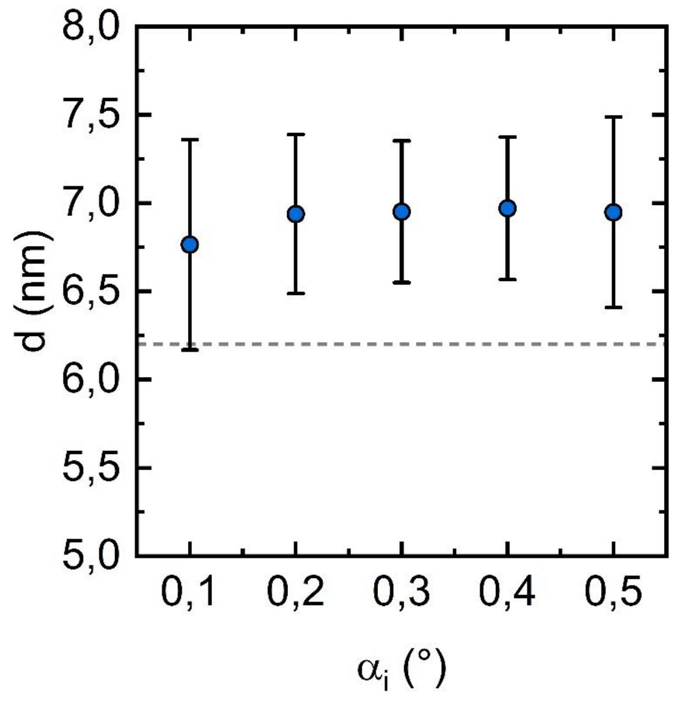

Figure S9. In-plane spacing of CQDs in a 130 nm EDA-treated SL as a function of incident angle in the x-ray scattering pattern. The shallower angles lead to less penetration of radiation while at higher angles the x-rays penetrate a significant or complete volume of the sample. Error bars represent the FWHM of the fitted peaks with a Pseudo-Voigt function. The grey dashed line indicates the CQD diameter. An invariance in the spacing as a function of depth in the sample demonstrates that the LE is complete throughout the whole thickness of the sample.

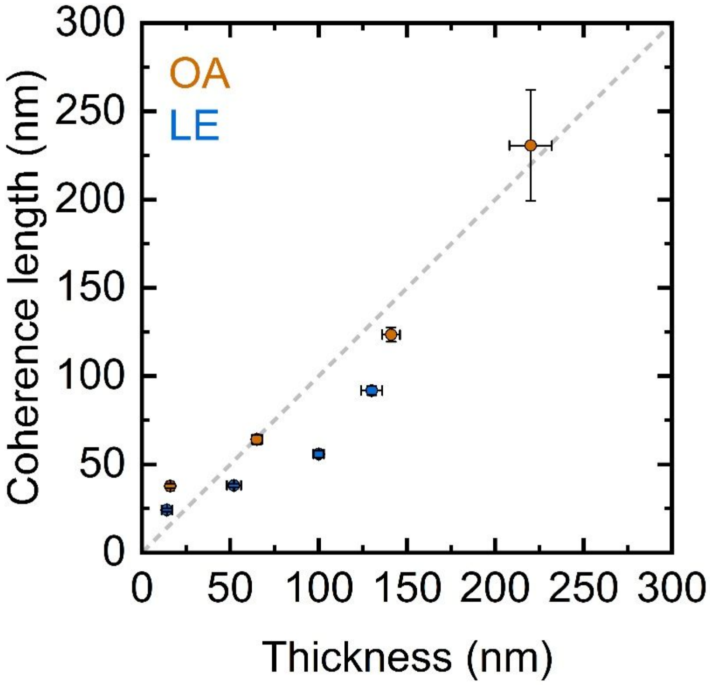

Figure S10. Measured out-of-plane coherence length as a function of thickness for SLs with oleic acid or EDA-treated. The grey dashed line indicates corresponding values. It can be noted that the OA samples are always close to being fully coherent along the thickness. The LE instead is partially detrimental to the coherence length. The values of the 3-layer samples are above the line which would be physically impossible. Besides sample-to-sample variation in thickness, the rational explanation is that the coherence length along *z* could be overestimated because the coherence lengths along *x* and *y* are very high. This is possible because we are considering the line cuts through the $(110)_{SL}$ direction which is parallel to the substrate.

Areal capacitance measurements

To determine accurately the areal capacitance of the ion gel-gated FETs (IGFETs), we measured its dependence on frequency and gate voltage using impedance spectroscopy. The main results of this analysis are reported in Figure S11. We used different methods to measure the capacitance: as a function of frequency and area, analogously to what we previously reported;[1,2] as a function of the applied DC gate voltage.[3] For the first method, we measured several devices that were characterized also as an IGFET, therefore comprising drain-source electrodes, PbS CQDs SLs, ion gel, and the gate electrode (Pt/PbS-SL/IG/Pt). Furthermore, we fabricated simpler structures comprising only ion gel in between the electrodes (ITO/IG/Pt). On all the devices, capacitance-frequency measurements were acquired through impedance spectroscopy setting a 200 mV AC voltage and no DC voltage applied to the top Pt gate. A characteristic curve can be seen in Figure S11a. The experimental data were fitted with an R-CPE model and the effective capacitance (i.e. capacitance at a specific frequency that takes into account the non-ideality of the ion gel as a capacitor) was calculated with the formula:

$$C_{eff} = Q(2\pi f)^{a-1} sin(-\pi a/2) \quad \text{(S1)}$$

Where Q is a frequency-dependent capacitance parameter, a is a parameter that quantifies the deviation from ideal capacitor behavior and f is frequency. As in our previous work, the transfer curves were measured at a rate of 5.7 mV/s which corresponds to a frequency of 570 mHz. The obtained effective capacitances were then plotted as a function of the effective area of the devices which was calculated with the formula:

$$A_{eff}^{-1} = A_{Pt}^{-1} + A_{IG}^{-1} \quad \text{(S2)}$$

Where A_{eff}^{-1} is the effective area, A_{Pt}^{-1} is the area of the platinum foil used as a gate electrode and A_{IG}^{-1} is the area covered by the ion gel droplet on the devices. The effective area is needed to consider the electrodes' geometry: since the gate has comparable dimensions to the device area, not all the voltage drops at the electrolyte-semiconductor (the PbS SL in our case) interface.[4] From the so-obtained C_{eff} vs. A_{eff} plot, the areal capacitance was determined with a linear fit fixing the intercept at the origin of the axes (Figure S11b). The measurements show that the capacitance of devices with or without the SL is comparable. The obtained areal capacitance of 4.7 μFcm^{-2} is in the expected range for such an ion gel and can be considered a reliable estimation for devices fabricated analogously.

We also performed a capacitance-frequency measurement with a 1.5 V DC voltage applied to the gate electrode and a 20 mV AC signal to test the effect of static gate bias (Figure S11c). We modeled this data with the R-CPE and we observed comparable areal capacitances at the frequency of interest.

To verify the dependence on the applied gate voltage, which is quasi-DC, we performed a capacitance-voltage measurement on a device with a 52 nm thick SL. The DC gate voltage was swept between -0.4 and 2 Volts with an applied 50 mV AC voltage at a frequency of 570 mHz. As can be seen in Figure S11d, the areal capacitance increases monotonically with the applied gate voltage and it reaches a comparable value as in Figure S11c. We estimated the accumulated carrier density as a function of gate voltage with the formula:

$$n(V_G) = \frac{1}{e} \cdot \int_0^{V_G} C(V_G) \cdot dV_G \quad \text{(S3)}$$

Where e is the elementary charge. The maximum carrier density accumulated at 2 V is around $2.15 \cdot 10^{13}$ cm^{-2}. This corresponds to an areal capacitance of about 4.6±0.3 μFcm^{-2}. The capacitance values at high gate voltage are comparable with the ones obtained from frequency sweeps confirming the result obtained. Contrary to a recent report that showed a strong dependence of the capacitance on the gate voltage for 2D PbS SLs with signs of band-filling, we do not observe this effect.[3] Overall we measure a lower capacitance which can be due to the presence of the polymeric matrix and the electrodes' geometry.

Since the two methods agree with each other and give comparable results, we consider the capacitance obtained with the same method as in our previous works for ease of comparison.

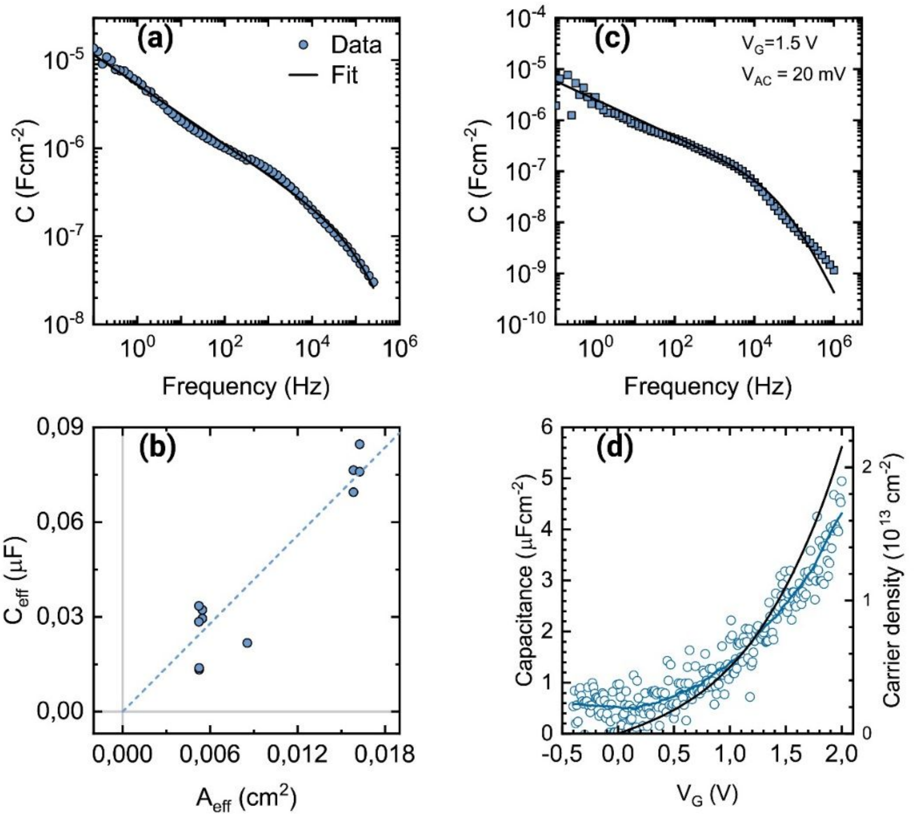

Figure S11. Determination of areal capacitance in the IGFETs. **(a)** Areal capacitance vs. frequency plot of an exemplary device comprising the PbS CQD superlattice. The continuous black line represents the best-fit curve with a R-CPE model. **(b)** Effective capacitance vs. effective area plot for a variety of devices including the superlattice film (smaller effective areas) and without (larger effective areas). The dashed blue line is a linear fit with the intercept fixed at the origin used to determine the average areal capacitance in the IGFETs. **(c)** Analogous measurement as in (a) but with a DC voltage of 1.5 V applied to the gate as well as a 20 mV AC signal. The fitting was performed as in (a). **(d)** Areal capacitance plot as a function of the applied DC gate voltage in a device comprising the superlattice film (left axis). The curve has been measured with a frequency of 570 mHz and an AC signal of 50 mV amplitude. The scatter points represent the measured data while the continuous blue line is an adjacent-average smoothing for ease of visualization. Integrating this curve for positive voltages gives the accumulated carrier density in the transistor channel as a function of gate bias (black continuous line, right axis).

Thickness-dependent mobility

Among the set of devices with 30-minute LE, we compare the mobility of SLs with two different thicknesses (Figure S12), namely 52 and 100 nm. We performed a two-sample t-test with a confidence level of $\alpha>0.05$ and we found that the two distributions are significantly different ($\alpha=0.03$) with the thinnest SL having the highest mobilities. Since our devices have a top-gate bottom-contact geometry, the difference in performance could be explained by the longer path that electrons must travel to reach the electrodes in the thickest films. This translates to a higher effective channel length or a higher contact resistance. While this observation seems trivial, it might have a more important implication relating to the thickness of the accumulation layer. It is not clear to what extent the ion gel can penetrate in such 3D SLs whereas, in the 2D ones, it is assumed that all the charge is accumulated in the only QD layer present. If the ion gel was able to penetrate above 50 and up to 100 nm, the charge would be accumulated throughout the whole volume and the capacitance would increase linearly with thickness. Instead, our measurements indicate that thinner devices have higher currents, and the accumulation layer is probably limited to the superficial part of the SLs. This observation agrees with the lack of band-filling evidence, both in the transfer curves and the C-VG measurements. We believe that this is evidence that the ion gel does not penetrate significantly in 3D QD SLs, but more in-depth experiments and more extensive statistics are required to further prove this point.

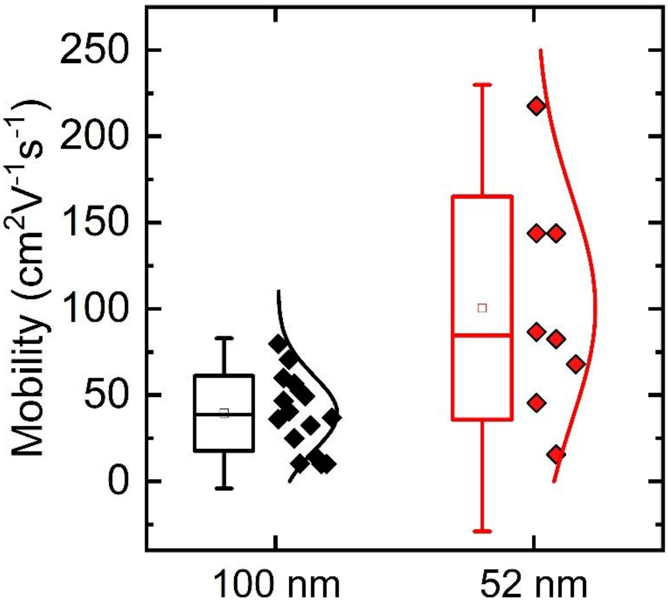

Figure S12. Comparison of mobility distributions in samples of 100 and 52 nm thick with a 30-minute ligand exchange.

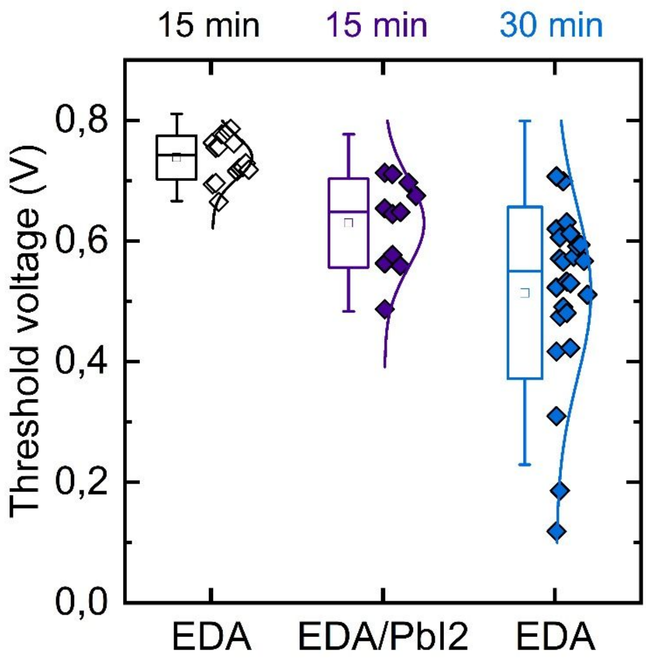

Figure S13. Comparison of the threshold voltage distributions for the three different treatments presented in the main text.

Supplementary references

(1) Balazs, D. M.; Matysiak, B. M.; Momand, J.; Shulga, A. G.; Ibáñez, M.; Kovalenko, M. V.; Kooi, B. J.; Loi, M. A. Electron Mobility of 24 $cm^2 V^{-1} S^{-1}$ in PbSe Colloidal-Quantum-Dot Superlattices. *Advanced Materials* **2018**, *30* (38).

(2) Pinna, J.; Koushki, R. M.; Gavhane, D. S.; Ahmadi, M.; Mutalik, S.; Zohaib, M.; Protesescu, L.; Kooi, B. J.; Portale, G.; Loi, M. A. Approaching Bulk Mobility in PbSe Colloidal Quantum Dots 3D Superlattices. *Advanced Materials* **2023**, *35* (8), 2207364.

(3) Septianto, R. D.; Miranti, R.; Kikitsu, T.; Hikima, T.; Hashizume, D.; Matsushita, N.; Iwasa, Y.; Bisri, S. Z. Enabling Metallic Behaviour in Two-Dimensional Superlattice of Semiconductor Colloidal Quantum Dots. *Nature Communications 2023 14:1* **2023**, *14* (1), 1–10.

(4) Gutiérrez-Lezama, I.; Ubrig, N.; Ponomarev, E.; Morpurgo, A. F. Ionic Gate Spectroscopy of 2D Semiconductors. *Nature Reviews Physics 2021 3:7* **2021**, *3* (7), 508–519.
